# A Closed-Loop Model of the Respiratory System: Focus on Hypercapnia and Active Expiration

**DOI:** 10.1371/journal.pone.0109894

**Published:** 2014-10-10

**Authors:** Yaroslav I. Molkov, Natalia A. Shevtsova, Choongseok Park, Alona Ben-Tal, Jeffrey C. Smith, Jonathan E. Rubin, Ilya A. Rybak

**Affiliations:** 1 Department of Mathematical Sciences, Indiana University - Purdue University, Indianapolis, Indiana, United States of America; 2 Department of Neurobiology and Anatomy, Drexel University College of Medicine, Philadelphia, Pennsylvania, United States of America; 3 Department of Mathematics, University of Pittsburgh, Pittsburgh, Pennsylvania, United States of America; 4 Institute of Information and Mathematical Sciences, Massey University, Albany, Auckland, New Zealand; 5 Cellular and Systems Neurobiology Section, National Institute of Neurological Disorders and Stroke, National Institutes of Health, Bethesda, Maryland, United States of America; Emory University School of Medicine, United States of America

## Abstract

Breathing is a vital process providing the exchange of gases between the lungs and atmosphere. During quiet breathing, pumping air from the lungs is mostly performed by contraction of the diaphragm during inspiration, and muscle contraction during expiration does not play a significant role in ventilation. In contrast, during intense exercise or severe hypercapnia forced or active expiration occurs in which the abdominal “expiratory” muscles become actively involved in breathing. The mechanisms of this transition remain unknown. To study these mechanisms, we developed a computational model of the closed-loop respiratory system that describes the brainstem respiratory network controlling the pulmonary subsystem representing lung biomechanics and gas (O_2_ and CO_2_) exchange and transport. The lung subsystem provides two types of feedback to the neural subsystem: a mechanical one from pulmonary stretch receptors and a chemical one from central chemoreceptors. The neural component of the model simulates the respiratory network that includes several interacting respiratory neuron types within the Bötzinger and pre-Bötzinger complexes, as well as the retrotrapezoid nucleus/parafacial respiratory group (RTN/pFRG) representing the central chemoreception module targeted by chemical feedback. The RTN/pFRG compartment contains an independent neural generator that is activated at an increased CO_2_ level and controls the abdominal motor output. The lung volume is controlled by two pumps, a major one driven by the diaphragm and an additional one activated by abdominal muscles and involved in active expiration. The model represents the first attempt to model the transition from quiet breathing to breathing with active expiration. The model suggests that the closed-loop respiratory control system switches to active expiration via a quantal acceleration of expiratory activity, when increases in breathing rate and phrenic amplitude no longer provide sufficient ventilation. The model can be used for simulation of closed-loop control of breathing under different conditions including respiratory disorders.

## Introduction

The neural control of ventilation in mammals serves two major functions. First, it establishes the automatic rhythm that produces periodic contraction of respiratory muscles, resulting in rhythmic inflation and deflation of the lungs that underlie the exchange of gases between the lungs and air. Second, it adjusts the rhythm and pattern of breathing to current metabolic demands (defined by the levels of carbon dioxide and oxygen in blood and tissues) and mechanical conditions (execution of various movements, maintenance of posture, and so on) as well as to various non-ventilatory behaviors (such as speaking, sniffing, and eating). The basic respiratory rhythm is generated by special neural circuits that are functionally and spatially organized within the brainstem and represent the respiratory central pattern generator (CPG) [1]–[6]. Like other CPGs, the brainstem respiratory CPG is capable of autonomously generating rhythmic activity without receiving patterned or periodic external inputs or feedback signals. Nevertheless, the operation of this CPG is controlled by various descending and afferent feedback signals. There are two major types of feedback that control the respiratory CPG: mechanical feedback, provided by pulmonary mechanoreceptors carrying information on lung volume, and chemical feedback from peripheral and central chemoreceptors, informing the CPG about the levels of carbon dioxide and oxygen in the blood and brain tissue [1], [2]. In turn, the feedforward neural connections from the CPG involve the bulbospinal neurons in the brainstem projecting to phrenic motoneurons in the spinal cord that control rhythmic contractions of the diaphragm (the principal respiratory muscle) and to other motoneurons that control abdominal and intercostal muscles. The latter can, in some conditions, be involved in active expiration by contributing to pumping air out of the lungs.

Previous models of the respiratory system (e.g., [7]–[19], see also a recent review [20]) described this system at different levels of details with major focus on the neural controller (for example, [10], [12], [18], [19]), on lung mechanics (for example, [17], [19]), or on gas exchange (for example, [7], [9], [15], [16]), but did not consider all three components of this closed-loop system together with the account of both mechanical and chemical feedback from the lungs to the neural controller. Moreover, the previous models focused mainly on quiet (passive) breathing, during which the pumping of air in the lungs is mostly performed by the diaphragm, whose contraction is controlled by the CPG during inspiration. During such quiet breathing, muscle contraction during expiration is minimal and does not play a significant role in ventilation. In contrast, during intense exercise or severe hypercapnia forced or active expiration occurs [21]–[23]. In such cases, the abdominal “expiratory” muscles become actively involved in breathing and ventilation by providing a forced expulsion of the air from the lungs during expiration. Within the CPG, the transition from quiet breathing to breathing with active or forced expiration is accompanied by emerging phasic activity [24], [25] connected with activation of an additional neural oscillator representing the so-called parafacial respiratory group (pFRG) located within, or overlapping with, the retrotrapezoid nucleus (RTN) [26]–[31]. The emergence of RTN/pFRG oscillations causes well-expressed pre-inspiratory (pre-I) or late-expiratory (late-E) discharges in the abdominal motor output, which appear at the end of expiration just before, and coupled to, the inspiratory bursts in the phrenic nerve [26]–[29]. It has been proposed that the RTN/pFRG generator driving the abdominal late-E discharges is activated by central chemoreceptors sensing an increase in concentration of carbon dioxide during hypercapnia [28], [29], [32], [33]. However, the exact role of abdominal late-E discharges in the framework of the closed-loop respiratory control system and how these discharges affect ventilation have not been previously considered.

The main objectives of this study were: (1) to develop and analyze a closed-loop computational model of the neural control of ventilation with well elaborated models of both the neural controller (brainstem respiratory network) and the pulmonary components (lung mechanics and gas exchange) providing both lung volume-dependent mechanoreceptor and CO_2_-dependent central chemoreceptor feedback to the neural controller and (2) to investigate the mechanisms of the closed-loop control of ventilation during the transition from quiet breathing to breathing with forced or active expiration with the development of hypercapnia.

Based on our simulations we suggest that the closed-loop respiratory control system switches to active or forced expiration when further increases of ventilation by simple increases in the rate of breathing and phrenic amplitude (defining the maximal lung inflation) become insufficient for providing gas homeostasis in the blood and tissue. In such cases, the control system activates the additional abdominal pump controlled by the late-E activity that emerges in the RTN/pFRG and provides an additional expulsion of air from the lungs by reducing the minimal lung volume, thereby permitting larger tidal volumes for augmented ventilation.

## Model

### The Closed-loop Control of Breathing: System Organization and Interactions

**Figure 1:**
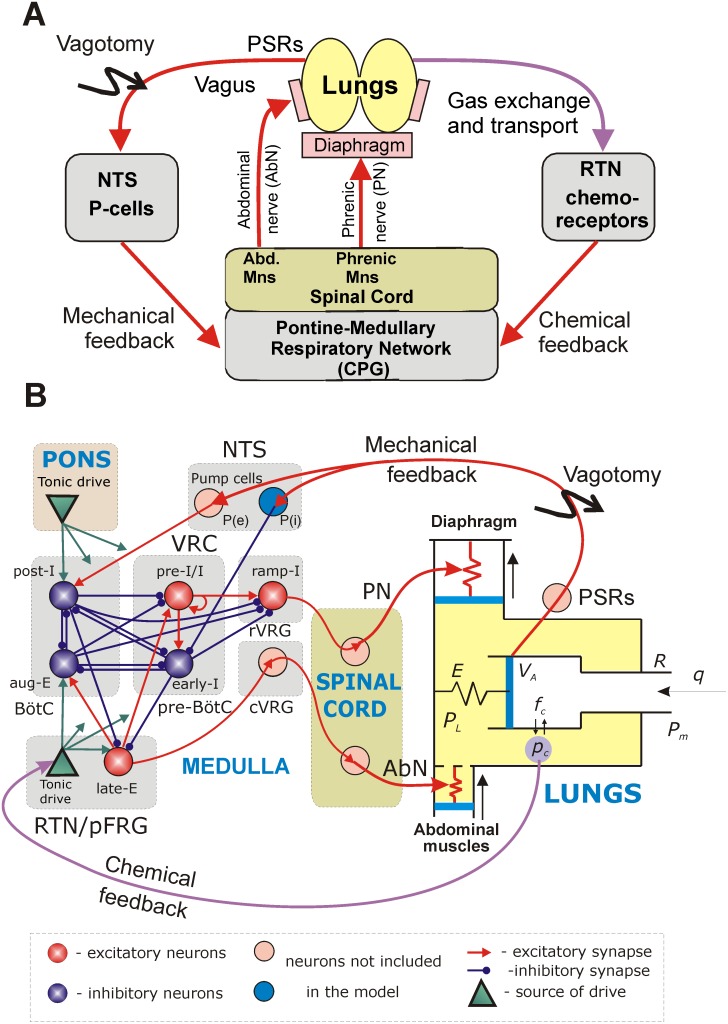
Closed-loop respiratory system. **A**. General diagram of interactions between the brainstem respiratory neural network and the lungs. **B**. Model schematic. See text for detailed description. ***Abbreviations*** **:** Abd. – abdominal; AbN – abdominal nerve; aug-E – augmenting expiratory neuron; BötC – Bötzinger complex; CPG – central pattern generator; cVRG – caudal ventral respiratory group; early-I – early-inspiratory neuron; late-E – late-expiratory neuron; Mns – motoneurons; NTS - nucleus of the tractus solitarius; P-cells – Pump cells; P(e) – excitatory pump cells; P(i) – inhibitory pump cells; pFRG – parafacial respiratory group; PN – phrenic nerve; post-I – post-inspiratory neuron; pre-BötC – pre-Bötzinger complex; pre-I/I – pre-inspiratory/inspiratory neuron; PSRs – pulmonary stretch receptors; ramp-I – ramp-inspiratory neuron; RTN – retrotrapezoid nucleus; rVRG – rostral ventral respiratory group; VRC – ventral respiratory column.

We developed a mathematical model of the closed-loop system for the control of breathing, as illustrated in a general diagram in Figure 1A. Breathing or lung ventilation represents the exchange of air between the lungs and air. This exchange is provided by the rhythmic contraction of the diaphragm and other (abdominal and intercostal) muscles, which in turn are controlled by the corresponding pools of motoneurons whose activities are conveyed by the corresponding nerves (phrenic, abdominal, and so on). The firing activities of the phrenic and abdominal motoneurons represent major motor outputs of the respiratory network that contains the respiratory CPG generating respiratory oscillations. There are two major feedback pathways from the lungs to the brainstem respiratory CPG. First, mechanical feedback is provided by pulmonary stretch receptors (PSRs) that are located in the lungs and transmit information on the lung volume. The PSR axons enter the brainstem in the vagus nerve and activate the so-called 2^nd^-order or pump (P) cells in the nucleus of the solitary tract (NTS) (see Figure 1A, B). Second, chemical feedback is provided by peripheral and central chemoreceptors, whose activity is sensitive to the levels of oxygen and carbon dioxide in the blood and brain tissue (only the pathway for CO_2_-dependent central chemoreceptor feedback was modeled as shown in Figure 1). According to recent studies [34], [35], a significant contribution to the function of central chemoreception arises from RTN neurons, whose activity is highly sensitive to CO_2_ concentration in the brainstem and which provide a CO_2_-dependent excitatory drive to the brainstem respiratory network (Figure 1A, B).

### Respiratory Network and the Respiratory CPG

The model of the brainstem respiratory network is based on the previous models of the respiratory CPG developed by Rubin et al. [36], [37], which represent reduced versions of previous large-scale models [3], [4], [12], [29]. The present model describes neural circuits in the ventral respiratory column (VRC) interacting with other brainstem compartments including the pons, NTS and RTN/pFRG (Figure 1B). The VRC consists of three compartments (left-to-right in Figure 1B): the Bötzinger Complex (BötC), the pre-Bötzinger Complex (pre-BötC), and the rostral ventral respiratory group (rVRG). The BötC contains two major types of inhibitory expiratory neurons, post-inspiratory (post-I) and augmenting-expiratory (aug-E); the pre-BötC includes the major inspiratory neuron types: pre-inspiratory-inspiratory (pre-I/I) and early-inspiratory (early-I), and the rVRG contains the major premotor (ramp-inspiratory, ramp-I) neurons projecting to phrenic motoneurons (not included in the model) controlling the diaphragm (Figure 1B). There is also a separate caudal VRG (cVRG) compartment containing bulbospinal expiratory neurons.

**Figure 2:**
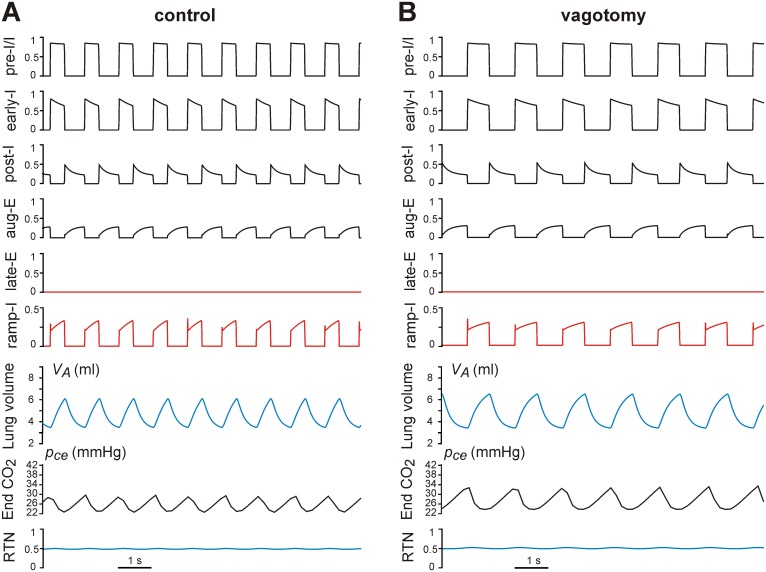
Model performance in control conditions (A) and after vagotomy (B). The top 6 traces in **A** and **B** represent output activity (normalized firing rate) of the corresponding neurons. The bottom two traces represent the end capillary blood *p_c_* (*p_ce_*) just before the next heart beat and the RTN drive, respectively. Note that vagotomy (removal of mechanical feedback) prolongs inspiration and expiration, increases the amplitude of ramp-I (and hence PN) activity and lung inflation (maximal lung volume and tidal volume), and slows the respiratory oscillations.

Synaptic interactions between these neuron types within the VRC, as shown in Figure 1B, were described in detail and justified earlier [36], [37]. Generation of the normal rhythmic respiratory activity is based on the interactions between a ring-like inhibitory network comprised of the post-I, aug-E, and early-I neurons and the excitatory pre-I/I neurons. The latter neurons have intrinsic bursting properties defined by the persistent sodium current (*I*_NaP_). They dynamically participate in the expiratory–inspiratory phase transition and maintain the inspiratory activity. The behavior of this network depends on, and is defined by, the excitatory drives from the pons and RTN/pFRG (Figure 1B). Interactions between the late-E neurons of the RTN/pFRG and the core neurons in the BötC and pre-BötC follow the circuit organization proposed and justified in several previous studies [28], [29], [37]. These interactions include: (a) excitation of the pre-I/I inspiratory neurons of the pre-BötC and the aug-E neurons of the BötC by the late-E neurons and (b) inhibition of the late-E neurons by the early-I neurons of the pre-BötC during inspiration and by the post-I neurons of the BötC during expiration (see Figure 2B). The rhythmic activity emerging in the late-E neurons of the RTN/pFRG under certain conditions (e.g., during hypercapnia) is defined by a *I*_NaP_-dependent mechanism [29].

All neurons were simulated by an activity-based (non-spiking) neuron model. This simplified yet well-established general form of neuronal model was used in our previous models of the brainstem respiratory network [36], [37]. These modeling studies have demonstrated that this simplified description provides a sufficiently rich representation of the neural dynamics to capture experimentally observed phase transitions and responses to a variety of perturbations, such that a more complex spiking formulation is not necessary. Each neuron in the present model represents a specific neural population described in an activity-based framework, in which the dependent variable *V* represents an average voltage for the neural population and each output *f*(*V*) represents the average or integrated population activity (firing rate) at the corresponding average voltage [36]–[38]. For the pre-I/I and late-E neurons, this simplified description includes the explicit representation of *I*_NaP_, which allows us to consider the role of *I*_NaP_ in these populations in generating rhythmic activity.

### Lung Mechanics and Gas Exchange and Transport

The lung and gas exchange sub-system in the model is based on the earlier models of Ben-Tal and Smith [15], [16]. In the present study, parameter values for this sub-system were chosen from the physiological literature to represent the pulmonary system of an adult rat. Similar to the previous models, the lungs are considered as a single container. In contrast to the previous models, which contained only one pump simulating the effects of diaphragm contractions, the present model contains two pumps, one representing the diaphragm and the other simulating the effects of abdominal muscle contractions (Figure 1B). Both the diaphragm and the abdominal muscles are modeled as springs, whose lengths are controlled by the external forces defined by the activities of the corresponding phrenic (PN) or abdominal (AbN) nerves, respectively. Changes in the muscle lengths cause changes in the pleural pressure *P_L_*, which decreases with contraction of the diaphragm and increases with contraction of the abdominal muscles. In the absence of any inputs to the respiratory muscles there is a negative pressure difference -*P_Lo_* between the pleural pressure and the mouth pressure that (together with the lung elastance) defines the lungs’ functional residual capacity. The pleural pressure affects the alveolar pressure *P_A_*. The difference between the alveolar pressure and the pleural pressure, divided by the lung elastance, defines the lung volume *V_A_*.

To model gas exchange and gas transport, it is assumed that the volume of the capillaries is the same as the heart stroke volume and that the transit time of blood through the lung is the same as the time interval between heart beats. Movement of blood through the lungs is simulated by re-initializing the values of blood partial pressures of carbon dioxide and oxygen on each heart beat (for more details see [39]). The heart is modeled as a pump that at every heart beat instantaneously delivers a portion of the venous blood with preset partial pressures of oxygen *p_o_* = 39 mmHg and carbon dioxide *p_c_* = 45 mmHg. Between heart beats, these pressures are adjusted due to diffusion between the blood and alveoli. The values of the partial pressures at the end of each interbeat interval are accepted as the arterial partial pressures of oxygen and carbon dioxide, *p_oe_* and *p_ce_*, respectively.

### Feedforward and Feedback Connections

For simplicity, the phrenic and abdominal motoneurons, and the premotor cVRG neurons have not been explicitly modeled. Instead, the output activities of the ramp-I and late-E neurons are used to represent phrenic and abdominal motor outputs, respectively. These signals implicitly define the forces causing the contraction of the diaphragm and abdominal muscles, respectively (Figure 1A, B).

A key new feature of the present model is the inclusion of two feedback types, mechanical and chemical. Mechanical feedback is normally provided by signals from PSRs, which respond to lung inflation [18]. Since PSRs have not been explicitly included in our model, the mechanical feedback signal is considered equal to the excess of lung volume above the functional residual capacity during inspiration (*V_insp_*) and equal to zero when the lung volume is below the functional residual capacity. This feedback is conveyed to the brainstem respiratory controller via the excitatory (P(e)) and inhibitory (P(i)) P-cells of the NTS (Figure 1). In the model, for simplicity, the mechanical feedback directly excites the post-I neuron and inhibits the early-I neuron (Figure 1B, see also [18]). Since the lung volume increases during inspiration, the mechanical feedback leads to a premature termination of the inspiratory phase and hence to an increase of breathing frequency. Vagotomy is modeled as removal of mechanical feedback by setting PSR activity to zero.

Chemical feedback in the model represents only central chemoreception and is provided via the CO_2_–dependent increase of tonic excitatory drive from the RTN to the respiratory network including the late-E neuron within the RTN/pFRG (Figure 1B). This drive is modeled as a saturating function of the low-pass filtered arterial CO_2_ partial pressure (*p_ce_*). An increase in the RTN drive largely affects the activity of the ramp-I neuron and thus amplifies the phrenic motor output, which increases the maximal lung volume. To a minor extent RTN output also modulates the frequency of breathing.

## Results

### Modeling the Effects of Vagotomy, Pontine Transection, and Vagal Stimulations

Respiratory oscillations emerge in the model within the BötC and pre-BötC compartments due to the dynamic interactions among the inhibitory early-I, post-I and aug-E neurons and the excitatory pre-I/I neurons (Figure 2A). A detailed description of these interactions can be found in our previous publications [3]–[6], [12]. In brief, during the expiratory phase of each oscillation, the activity of the inhibitory post-I neuron in BötC decreases because of its adaptation and increasing inhibition from the aug-E neuron (Figure 1B and Figure 2A, traces for post-I and aug-E). Eventually, the pre-I/I neuron of the pre-BötC escapes from the decreasing post-I inhibition and becomes active (Figure 2A, trace for pre-I/I), providing excitation to the inhibitory early-I neuron of the pre-BötC and the excitatory ramp-I neuron of the rVRG (Figure 1B). The early-I neuron then starts firing and inhibits the post-I and aug-E neurons. This leads to disinhibition of the inspiratory ramp-I neuron, completing the onset of inspiration.

During inspiration, the activity of the early-I neuron decreases due to adaptation (Figure 2A, early-I trace), which reduces the early-I inhibition of the BötC expiratory neurons. This decrease of inspiratory inhibition eventually becomes sufficient to allow activation of the post-I neuron. When the latter becomes active it inhibits all of the inspiratory neurons, providing an inspiratory off-switch and the onset of expiration (Figure 2A), and the whole cycle repeats. In the rVRG, the premotor ramp-I neuron receives excitation from the pre-I/I neuron, and its output activity represents the PN motor output. In turn, the early-I neuron shapes the augmenting pattern of the ramp-I neuron and hence the PN output.

Under normal (i.e. normocapnic) conditions, tonic drive to the late-E neuron of the RTN/pFRG is relatively weak and this neuron remains silent due to the inhibitory inputs from the post-I and early-I neurons (Figures 1B and 2A, B). Because of this inactivity, the AbN output during normocapnic conditions is equal to zero (Figure 1B). In turn, the PN output projects to the diaphragm (Figure 1B) producing diaphragm contraction and the corresponding lung inflation during inspiration (see the increase in the lung volume *V_A_* in each cycle in Figure 2A). Inspiration leads to the reduction of the alveolar partial pressure of CO_2_ (*f_c_*) and finally the partial pressure of CO_2_ in the blood (*p_c_*) (Figure 1B), which is reinitialized on each heart beat. The evolution of *p_c_* measured just before the onset of each heart beat (*p_ce_*) is shown in Figure 2A (see the trace for End CO_2_). The *p_ce_* value exhibits low-amplitude oscillations, decreasing during inspiration and increasing during expiration.

As seen in Figure 2A, under normal conditions, the respiratory network in the closed-loop model generates respiratory oscillations and exhibits the typical activity patterns of different respiratory neuron types, including an augmenting firing profile of the ramp-I neuron (and hence the PN output) during inspiration. In the control case, the duration and amplitude of the inspiratory activity and the frequency of respiratory oscillations are controlled by the mechanical feedback representing the so-called Hering-Breuer (HB) reflex that causes an advanced termination of inspiration [1], [40], [41]. Similar to our previous models [10], [12], [18], the HB reflex operates due to the excitatory influence of lung inflation on the post-I neuron (via PSRs and P(e)) and the inhibitory influence on the early-I neuron (via PSRs and P(i), see Figure 1B). Therefore, simulated vagotomy (removal of mechanical feedback) leads to the loss of the HB reflex resulting in prolonged inspiration, increased amplitude of PN output and lung inflation (maximal lung volume), and slowed respiratory oscillations (Figure 2B), all of which are consistent with the existing experimental data [1], [41].

**Figure 3:**
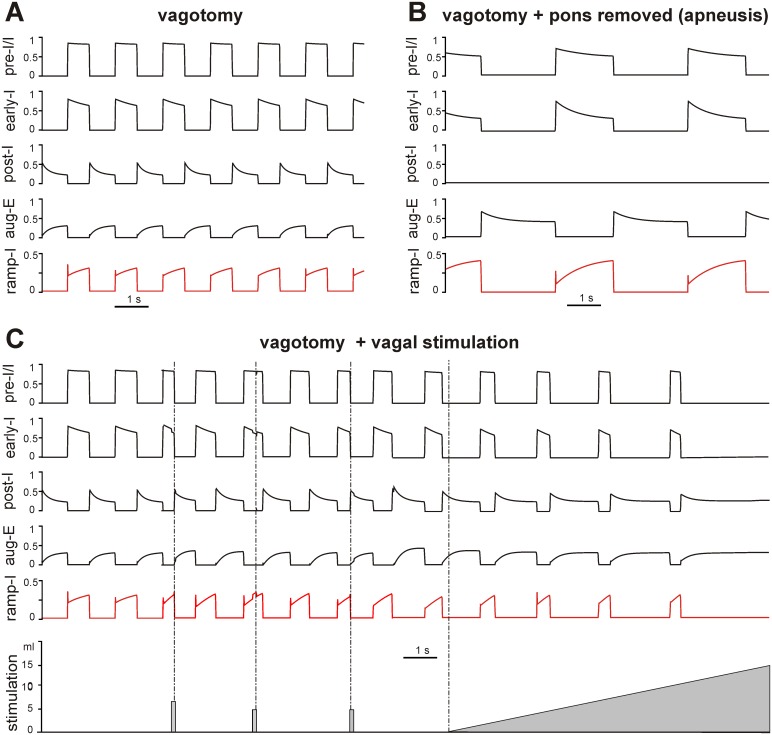
Perturbations of respiratory neural activity pattern by pontine removal and vagal stimulations. **A**. Model performance after vagotomy. **B**. Model performance after vagotomy and subsequent removal of pontine excitatory drive. Note the apneustic breathing pattern characterized by the significant increase in the duration of inspiration and slowing of the respiratory oscillations. **C**. Simulations of the effects of brief and continuous stimulation of mechanoreceptor afferents. The first stimulus (bottom trace, 7 ml of lung inflation) applied in the middle of the respiratory phase terminated the current inspiration. The second, reduced stimulus (5 ml) applied at the same phase was unable to terminate inspiration. The third stimulus of the same size as the second one (5 ml) applied later in inspiration terminated the inspiratory phase. Finally, continuous linearly increasing stimulation was applied. This stimulation shortened inspiration and prolonged expiration and then produced expiratory “apnea”, when all inspiratory neurons were inhibited by continuously active expiratory neurons.

Figure 3B shows an example of our simulation of the effect of pontine drive removal after vagotomy. Similar to our previous models [12], [18], the removal of the pontine input to the medullary circuits leads to an apneustic breathing pattern characterized by a significant increase in the duration of inspiration and a significant reduction of oscillation frequency (Figure 3B). The “apneustic” prolongation of inspiration results from the elimination of post-I activity due to the removal of pontine excitatory drive to this neuron and its inhibition by the aug-E neuron (Figures 1B and 3B). These features are typical for apneusis observed in experimental animals [1], [42]–[44].

Figure 3C shows the results of our simulations of the effect of brief and continuous stimulations of mechanoreceptor afferents (“vagal” stimulation). In these simulations, stimulation was applied either as brief pulses (100 ms) of lung volume (first three stimuli in Figure 3C, bottom trace) or as a continuous linear increase of lung volume over a 10 s interval (last stimulus in Figure 3C, bottom trace). Our simulations show that a brief vagal stimulation applied during inspiration can terminate the inspiratory phase and that the threshold for such inspiratory termination decreases during inspiration. Specifically in Figure 3C, the first stimulus (equivalent to 7 ml of lung inflation) applied in the middle of the inspiratory phase terminated the current inspiration; the second, reduced stimulus (5 ml) applied at the same phase was unable to terminate inspiration; and the third stimulus of the same size as the second one (5 ml) applied later in inspiration terminated the inspiratory phase. These simulation results are consistent with the existing experimental data [1], [40] and previous modeling studies [12]. Continuous stimulation (lung inflation) shortened inspiration and prolonged expiration, finally leading to expiratory “apnea”, during which all inspiratory neurons (and the PN) were inhibited by continuously active expiratory neurons, which is also consistent with the existing experimental data [1], [41], [45], [46].

### Modeling Hypercapnia and Active Expiration

As shown in many physiological studies, hypercapnia causes an increase of ventilation. Ventilation grows due to increases in both the amplitude (lung tidal volume) and the rate of breathing (respiratory frequency), at least when vagal feedback is intact [1], [47]–[50]. With the progressive development of hypercapnia (a significant increase of CO_2_ levels in blood and tissue), the respiratory system undergoes a transition from passive expiration typical for quiet breathing to breathing with active or forced expiration that involves recruitment of abdominal muscles contributing to pumping air out of the lungs [21]–[23]. Current experimental evidence indicates that these muscles are activated by the late-expiratory (late-E or pre-inspiratory) rhythmic discharges that emerge in the medullary RTN/pFRG region and drive expiratory bulbospinal premotor neurons [26]–[29]. These late-E discharges are coupled (phase locked) to the phrenic discharges. Similar to our previous models [28], [29], [37], the late-E discharges in the present model are generated by the late-E neuron located in the RTN/pFRG (see Figure 1B). The late-E neuron is activated when it receives sufficiently strong excitatory drive from the RTN, which is defined by the arterial CO_2_ partial pressure, *p_ce_*; that is, late-E activation occurs when *p_ce_* exceeds some threshold level. As shown previously [29], [37], with the progressive development of hypercapnia the late-E discharges appear coupled (phase locked) to phrenic discharges demonstrating a so-called *quantal acceleration* [29]; i.e., they appear periodically with some ratio to the phrenic bursts (1∶4, 1∶3, 1∶2). This ratio increases until it reaches the 1∶1 ratio for which each PN discharge is preceded by a late-E (and AbN) discharge [29], [37].

**Figure 4:**
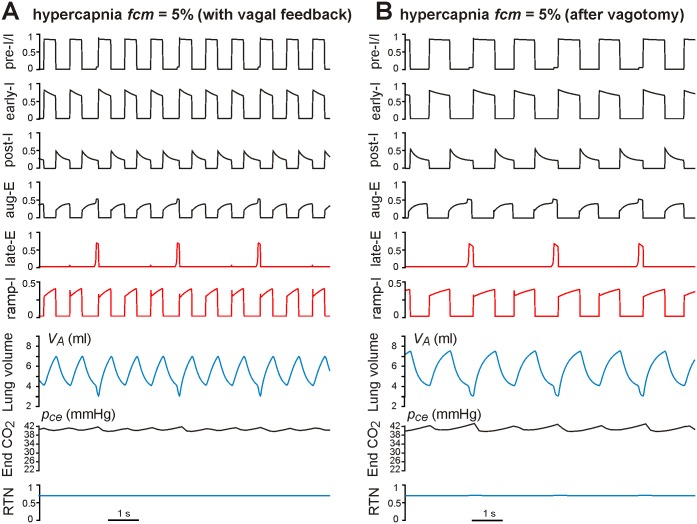
Effects of hypercapnia maintained at *f_cm_* = 5% in the intact (A) and vagotomized (B) models. *f_cm_* is CO_2_ content in the mouth. In both cases, hypercapnia evoked increases in RTN drive (bottom traces in **A** and **B**). The applied hypercapnia increased the RTN drive to the late-E neuron evoking late-E discharges with the ratio 1∶3 to the ramp-I discharges in the vagus intact model (late-E trace in **A**) and the ratio 1∶2 in the vagotomized model (late-E trace in **B**). Each late-E pulse actuates the abdominal pump, reducing the base level of lung volume (see V_A_ traces in **A** and **B**).

Hypercapnia in the model was simulated by assigning a value of the outside (within the mouth) CO_2_ concentration *f_cm_* (*f_cm_* = 0–10% with *f_cm_* = 0 representing normocapnia). The *f_cm_* level affects gas exchange dynamics and defines the arterial CO_2_ partial pressure, *p_ce_*, and finally the CO_2_-dependent RTN drive to both the VRC respiratory network and the late-E neuron within the RTN/pFRG (Figure 1B). The effects of sustained hypercapnia maintained at *f_cm_* = 5% on the neural activity pattern in the intact and “vagotomized” models are shown in Figure 4, panels A and B, respectively. In both cases, hypercapnia evoked increases in RTN drive (see bottom traces in Figure 4A, B and compare with the same traces in Figure 2A, B). The increased RTN drive to the VRC respiratory network resulted in increases in the amplitude and frequency of phrenic activity (PN, defined by the ramp-I activity) and, correspondingly, the amplitude (maximal value) and frequency of lung inflation (see *V_A_* traces in Figure 4A, B and compare with *V_A_* traces in Figure 2A, B). In addition, hypercapnia (at *f_cm_* = 5%) increased the RTN drive to the late-E neuron evoking late-E discharges with the ratio 1∶3 to the ramp-I (PN) discharges in the intact model (late-E trace in Figure 4A) and the ratio 1∶2 in the vagotomized model (late-E trace in Figure 4B). Each late-E pulse representing the activity of AbN (Figure 1B) actuates the abdominal pump to augment the pumping of air out of the lungs by reducing the basal (residual) level of the lung volume (see *V_A_* traces in Figure 4A, B), hence providing an additional increase of tidal volume and ventilation.

**Figure 5:**
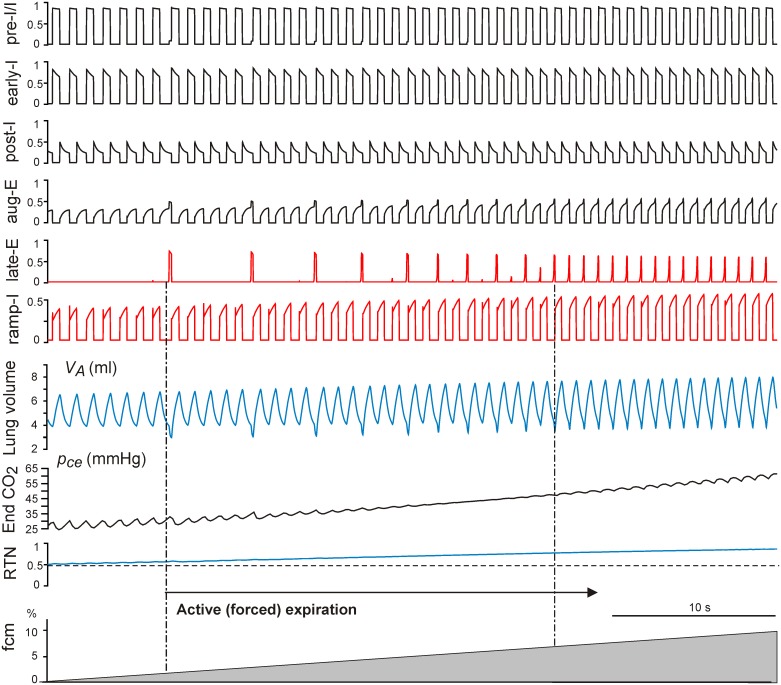
Simulation of progressive hypercapnia in the intact model. The continuous increase of hypercapnia (the grey ramp at the bottom) was induced by increasing CO_2_ content in the mouth *f_cm_* from 0 to 10%. Active expiration starts with the first appearance of late-E discharges (indicated by the left vertical dot-dashed line at *f_cm_* = 2.6%) and reaches the regime with a 1∶1 ratio of late-E to ramp-I activations at *f_cm_* = 7.2%. As described in the text, each late-E discharge, representing the activity of AbN output, actuates the abdominal pump that reduces the baseline level of lung volume (see in the V_A_ trace).

**Figure 6:**
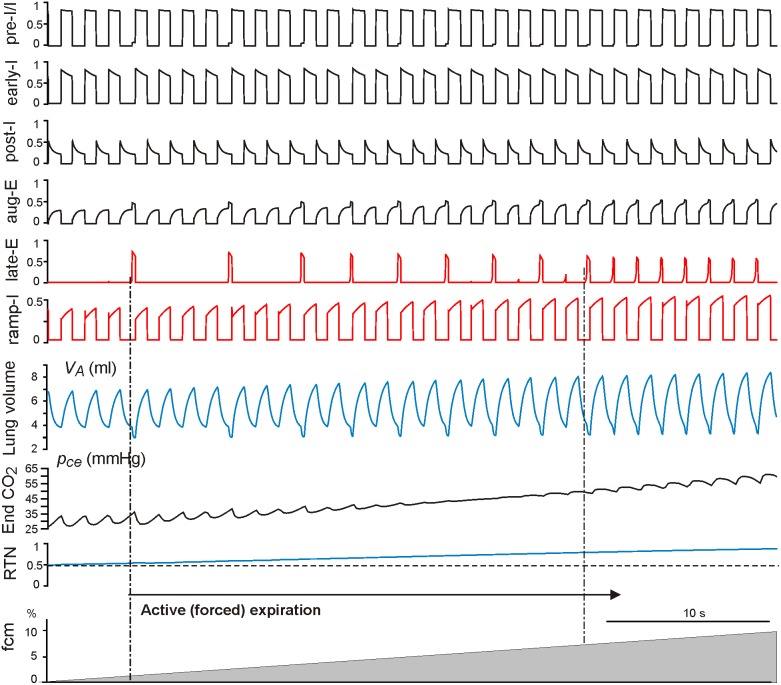
Simulation of progressive hypercapnia in the vagotomized model. The continuous increase of hypercapnia (the grey ramp at the bottom) was induced by increasing CO_2_ content in the mouth *f_cm_* from 0 to 10%. Active expiration starts with the first appearance of late-E discharges (indicated by the left vertical dot-dashed line at *f_cm_* = 1.2%) and reaches the regime with the 1∶1 ratio of late-E to ramp-I activations at *f_cm_* = 7%. Again, each late-E discharge, representing the activity of AbN output, actuates the abdominal pump that reduces the baseline level of lung volume (see in the V_A_ trace).

**Figure 7:**
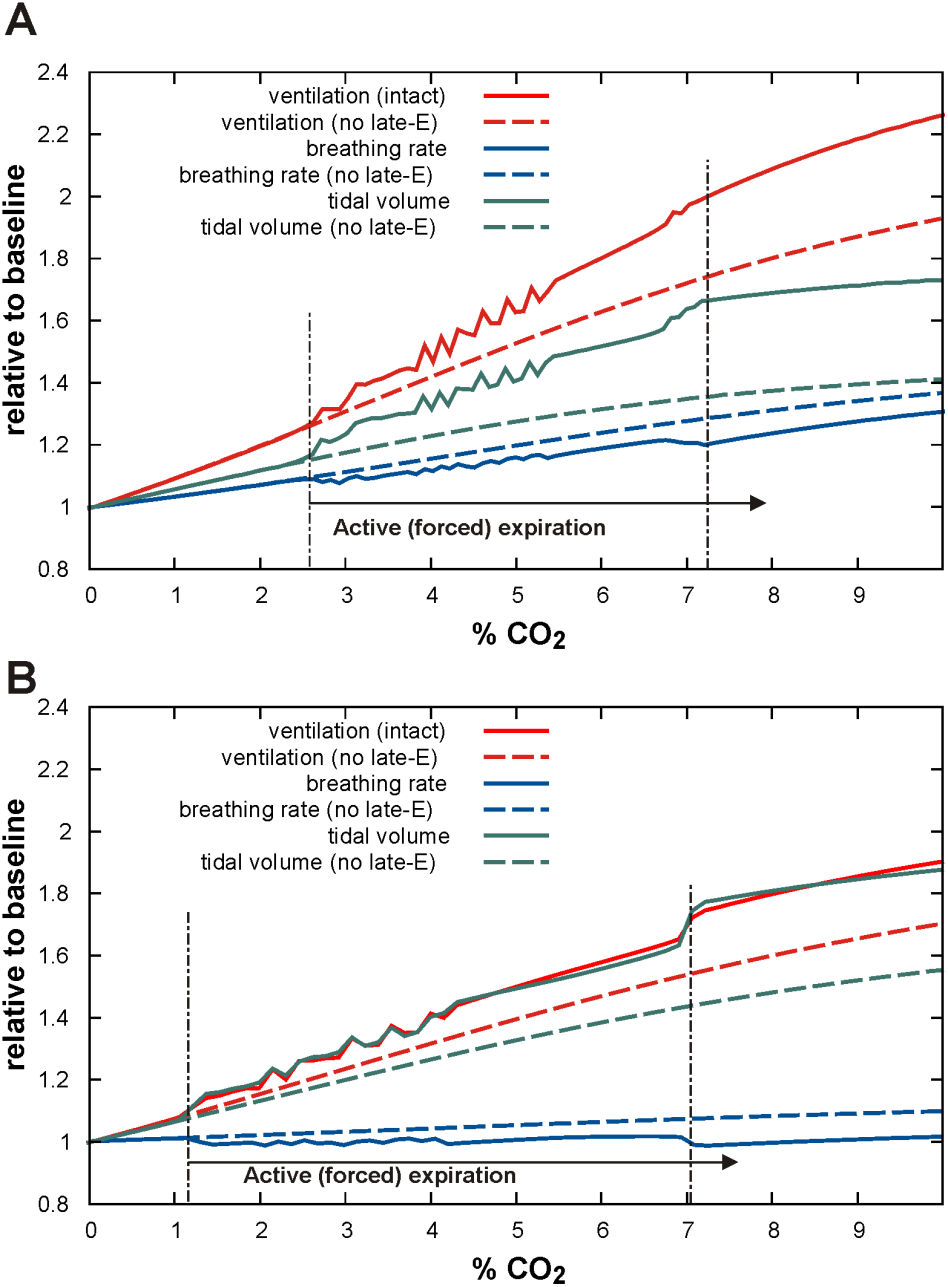
Changes in tidal volume, breathing rate, and ventilation (relative to normocapnia) with the development of hypercapnia in the intact (panel A) and vagotomized (panel B) models. CO2 content in the mouth *f_cm_* was linearly increased from zero to 10%. Changes in three major breathing characteristics (tidal volume, breathing rate, and ventilation) that would occur without active expiration (simulated by setting AbN = 0) are shown by the corresponding dashed lines. The vertical dot-dashed lines bound the development (quantal acceleration) of active expiration.

With the progressive increase of hypercapnia from *f_cm_* = 0 to *f_cm_* = 10% (grey triangle at the bottom of Figures 5 and 6), both the intact and vagotomized models show quantal acceleration of late-E activity leading to active (forced) expiration. As shown in Figures 5 and 6, in both cases, active expiration, indicated by a decrease in the minimal level of lung volume (see *V_A_* traces in Figures 5 and 6), starts with the first appearance of late-E discharges (indicated by the left vertical dot-dashed line at about *f_cm_* = 2.6% in Figure 5 and *f_cm_* = 1.2% in Figure 6, see also Figure 7AB). The regime with a 1∶1 ratio of late-E to PN discharges is reached at similar levels, namely *f_cm_* = 7.2% in the intact case (Figure 5 and 7A) and at *f_cm_* = 7% in the vagotomized case (Figure 6 and 7B).

As described above, each activation of the late-E neuron, and correspondingly each surge of AbN output (Figure 1B), actuates the abdominal pump, which reduces the basal level of lung volume. This reduction of basal level of lung volume provides an additional contribution to the increase of tidal volume and ventilation, relative to that observed in simulations without active expiration. Specifically, Figure 7A, B illustrates how tidal volume, breathing rate, and ventilation change relative to baseline levels (normocapnia) with the progressive development of hypercapnia (*f_cm_* increase from 0 to 10%) in the intact (panel A) and vagotomized (panel B) models, respectively. To visualize the role of active expiration, the changes of all three major characteristics (tidal volume, breathing rate, and ventilation) that would occur without active expiration (represented by setting AbN = 0) are also shown by the corresponding dashed lines. Our simulations demonstrate that active (forced) expiration provides a significant increase in ventilation in both intact and vagotomized cases. In both cases, the additional increase of ventilation (relative to passive ventilation without involvement of the late-E/AbN activity) results mainly from an increase of tidal volume, reflecting the amplitude of breathing, rather than an acceleration of breathing frequency. In fact, in the intact case, the increase in breathing rate with the development of hypercapnia is smaller with active expiration than with purely passive breathing (Figure 7A, blue lines), while in the vagotomized case, the breathing rate stays roughly constant and even exhibits small decreases when active expiration is present (Figure 7B, blue lines). Based on these simulations, we hypothesize that the closed-loop respiratory control system switches to active or forced expiration when further increases of ventilation via increases in the rate and amplitude of breathing become insufficient for providing gas homeostasis in the blood and associated tissue. In such cases, the control system automatically activates the abdominal pump controlled by the late-E activity emerging in the RTN/pFRG that provides additional exhalation of air from the lungs by reducing the minimal lung volume.

### Comparison of Model Simulations with Experimental Data

In the closed-loop control system considered here, the RTN represents the major site of CO_2_ central chemoreception [34], [35] and the target for chemical feedback. Therefore, one way to validate our model could be to study the effects of RTN activation on ventilation and its major characteristics in the model and to compare the results with the corresponding experimental data. Takakura et al. [51] reported that bilateral microinjection of substance P into the RTN in conscious rats increased breathing characteristics including the tidal volume, respiratory frequency, and ventilation. The results support the idea that RTN neurons activate facilitatory mechanisms important for the control of ventilation in resting, hypoxic or hypercapnic conditions in conscious rats [51].

**Figure 8:**
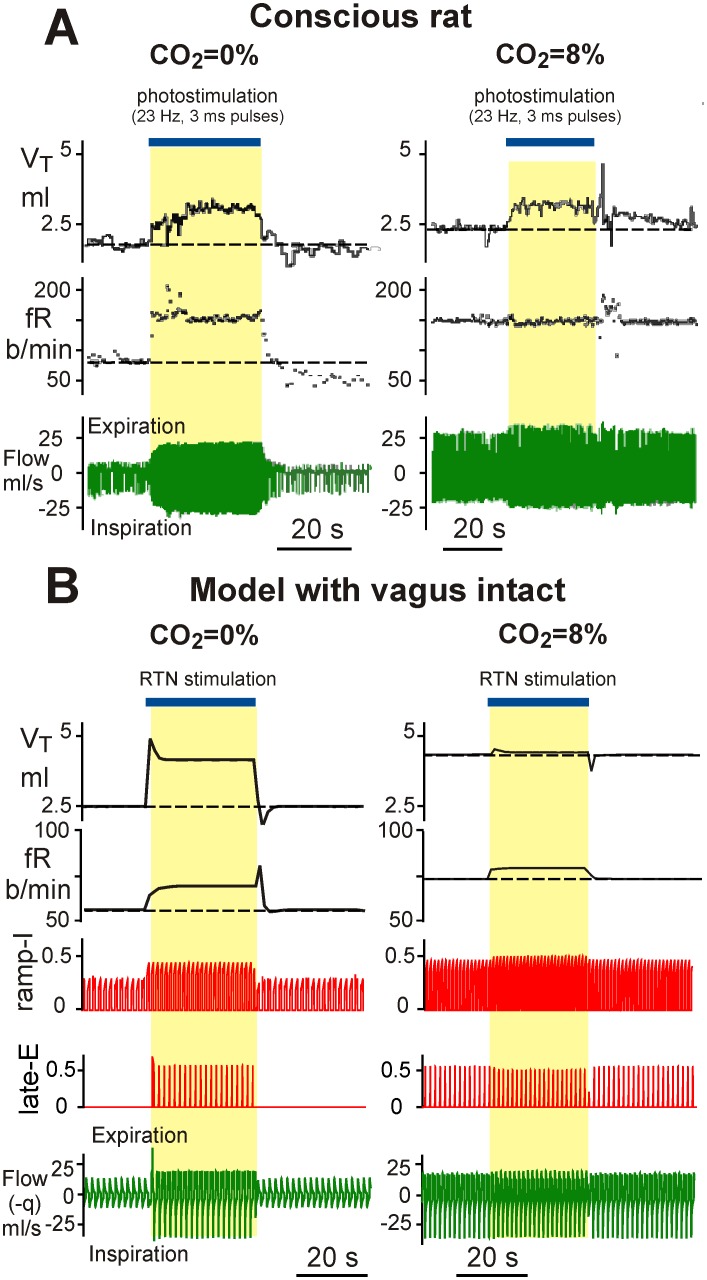
Comparison of model simulations with experimental data. **A**. Breathing stimulation elicited in conscious adult rat in a flow-through, whole-body plethysmography chamber by photostimulation via channelrodopsin genetically engineered in RTN Phox2b-expressing glutamatergic neurons. The left diagrams were constructed for hyperoxic normocapnia (100% O_2_), and the right diagrams for hypercapnia (8% CO_2_, balance O_2_). Continuous RTN stimulation (23 Hz, 3 ms pulses, 30 s total duration, blue bar at the top) raised tidal volume, V_T_, and breathing frequency, fR. During hyperoxic hypercapnia (right), RTN photostimulation produced a small increase in V_T_ but no increase in fR. Adapted from Abbott et al. [52], Fig. 1B, with permission. **B**. The results of our simulations. An additional 30s-duration increase in the blood CO_2_ level (Δ*p_c_*  = 30 mmHg, blue bar at the top) was applied in the normocapnic case (left) and on the background of simulated hypercapnia (*f_cm_* = 8%) (right).

Similar experiments in conscious rats were performed by Abbot et al. [52], who used optogenetic photostimulation of the key RTN glutamatergic neurons. Some of their results are shown in Figure 8A. In these experiments, the researchers performed photostimulation of the Phox2b-expressing glutamatergic neurons of the RTN in conscious rats during hyperoxic normocapnia (left) and hypercapnia (8% CO_2_, right). In normocapnic conditions (Figure 8A, left), this RTN stimulation produced significant increases in ventilation (bottom trace) and its major characteristics, the tidal volume and breathing frequency. In hypercapnic conditions (Figure 8A, right), this RTN stimulation produced a lesser effect on the tidal volume and ventilation with no influence on breathing frequency.

To simulate the above experiments with our model, we applied an additional 30s increase in the blood CO_2_ level (Δ*p_c_*  = 30 mmHg) in the normocapnic case (Figure 8B, left) and on the background of simulated hypercapnia (*f_cm_* = 8%) (Figure 8B, right). This additional increase of *p_c_* imitated stimulation of the RTN in the experimental data of Takakura et al. and Abbott et al. described above. Similar to the experimental studies, the applied RTN stimulation in normal conditions produced an increase in both the tidal lung volume and breathing frequency. Also, similar to the Abbot et al. experiments with RTN photostimulation in hypercapnic conditions, the model showed a significantly reduced amplification of both ventilation characteristics. The above similarities with experimental data provide an additional support for our model.

## Discussion

### Modeling the Closed-Loop Respiratory System

We have developed a novel closed-loop model of the respiratory system with well elaborated neural and pulmonary components: the brainstem respiratory network and CPG, representing the neural controller, and the biomechanical model of lungs including gas exchange and transport mechanisms, representing the controlled subsystem and providing both mechanical and chemical feedback to the neural controller. Previous models of this system did not consider all of these components together. For example, Rybak et al. [12] and later Molkov et al. [18] coupled a detailed description of the brainstem respiratory network to a simple lung model that provided only mechanical feedback from pulmonary stretch receptors to the respiratory network. The lung description in these models did not include lung mechanics, gas exchange and transport and chemical feedback. Longobardo et al. [14] adapted previously developed models of the respiratory CPG and connected them to a neurochemical feedback model to control ventilation. However, the model did not include lung mechanics and could not be used to study forced expiration. An integrated model of the respiratory system was proposed by Ben-Tal and Smith [15], [16]. The closed-loop model in these studies included lung mechanics and gas exchange (used as a basis for the model presented here). However, this model used a reduced description of the neural controller and did not include mechanical feedback from the lungs. Finally, O’Connor et al. [19] developed an advanced computational model of the respiratory neural network together with the biomechanics of breathing and airway defensive behavior. This model included a detailed representation of the respiratory neural network (based on interacting populations of integrate-and-fire neurons) connected with a well-elaborated, detailed biomechanical model of the lungs and upper airways. The objective of that study was to reproduce the respiratory motor pattern during eupneic breathing and its changes during several important motor behaviors, such as cough and swallow. Although this model had a more detailed description of the respiratory neural network and lung mechanics than what we consider, it did not incorporate gas exchange and transport components and did not include chemical feedback to the respiratory network, which would not allow this model to be used for simulating respiratory system behavior during hypercapnia and other metabolic challenges. Therefore, our work may represent the first model of the closed-loop respiratory system incorporating both mechanical and chemical feedback that simulates the transition to breathing with active expiration during hypercapnia. We extended the previously published model of lung mechanics and gas exchange [15] by incorporating an additional pump to simulate the effects of abdominal muscle contractions during active expiration We then connected this model with the previously developed, well elaborated model of the respiratory network [18], [37] with both mechanoreceptor and central chemoreceptor feedback. A more detailed and comprehensive model can be developed in the future by combining our model with the model of O’Connor et al. [19] and incorporating peripheral chemoreceptor feedback.

To test our model in a normal metabolic state (normocapnia) we performed several modeling experiments that simulated well-known experimental procedures including vagotomy (removing mechanoreceptor feedback), pontine transection, and phase-dependent brief and continuous stimulations of mechanoreceptor vagal feedback. Removal of vagal feedback in the model caused increases in the durations of both inspiration and expiration and in the maximum lung volume (*V_A_*) and lung tidal volume, along with a corresponding reduction in breathing frequency (Figure 2). These changes fully correspond to experimental data concerning the effects of vagotomy on the pattern of breathing [1], [41]. Furthermore, the subsequent removal of pontine drive to simulate pontine transection resulted in an “apneustic”-like output pattern (Figure 3B) characterized by significantly prolonged inspiration, which was typical for apneusis evoked by pontine transections in vagotomized animals [1], [42]–[44]. Our simulations of brief vagal stimulations (lung inflation) applied during inspiration (Figure 3C) confirmed that such stimulation could produce premature termination of inspiration and that the threshold for such inspiratory termination decreased during inspiration. These modeling results are also consistent with the existing experimental data [1], [40] and previous modeling studies [12]. Similarly, we showed that continuous vagal stimulation (lung inflation) produced shortened inspiration and prolonged expiration, and that increases in the strength of this stimulation lead to expiratory apnea, which is consistent with the existing experimental data as well [1], [41], [45], [46]. The results of these benchmark simulations provide an important validation of our closed-loop respiratory model, including the organization of interactions within the respiratory network and the organization and role of mechanoreceptor feedback in the closed-loop control of respiration.

### Modeling Hypercapnia and Active Expiration

The recruitment and activation of abdominal muscles during active expiration evoked by hypercapnia or exercise and their involvement in the amplification of ventilation have been amply documented [21]–[23]. The role of RTN/pFRG activation during hypercapnia leading to the generation of abdominal activity during active expiration has been also well described [26]–[29]. Nonetheless, no previous works have explicitly connected these two processes to consider how hypercapnia evokes RTN/pFRG activation and how the abdominal motor activity produced by RTN/pFRG activation affects ventilation in the context of a closed-loop control system. It appears that this study is the first to address these issues using a computer model of the closed-loop control of ventilation. To perform these investigations, we first modified the biomechanical lung model to incorporate an additional pump simulating the effect of abdominal muscle contractions on lung volume and ventilation (Figure 1B). Second, following previous models [32], [33] we simulated central chemoreceptor feedback, including a nonlinear dependence of RTN excitatory drive to the respiratory network and to the late-E neuron in the pFRG on the partial pressure of CO_2_ in blood and tissue, calculated in the model of the lungs with gas exchange and transport (Figure 1B). As a result, our model was able to simulate the behavior of the closed-loop respiratory system during hypercapnia and capture the transition to active expiration with an increase of CO_2_ level.

### Conclusions

Our simulations of hypercapnia predict that with the development of hypercapnia (associated with an increase in the external concentration of CO_2_) the respiratory system goes through a gradual transition to the regime of active expiration via the mechanism of quantal acceleration of the late-E and abdominal motor activity [29], [37]. This transition process is shown in Figures 5 and 6 for the intact and vagotomized cases, respectively. Based on our simulations we suggest that the additional increase in ventilation observed during severe hypercapnia occurs due to the quantally emerging late-E discharges in the RTN/pFRG and abdominal motor discharges phase-locked to the end of expiration (see Figure 7A, B). Specifically, abdominal motor outputs activate the abdominal pump and hence cause a reduction of basal lung volume and an increase in the amount of air expelled in each cycle in which they occur (see Figures 4–6). These effects lead to a significant increase in ventilation (Figure 7) that occurs due to a substantial enhancement of the “amplitude” of breathing (tidal volume) accompanied by a relatively small increase (intact case, Figure 7A) or even some decrease (vagotomized case, Figure 7B) in the breathing rate (respiratory frequency). Interestingly, the latter modeling-based conclusion is consistent with multiple experimental findings indicating that an increase in ventilation during hypercapnia in various preparations occurs mostly due to an increase in the breathing amplitude and that the respiratory rate does not significantly increase with hypercapnia after vagotomy [1], [47]–[50].

This work represents the first attempt to computationally model the closed-loop neural control of respiration in mammals with a specific focus on active expiration under conditions of hypercapnia. The model, although greatly simplified relative to the full complexity present biologically, reproduces multiple experimental phenomena related to mechanoreceptor and central chemoreceptor feedback to the neural controller in the brainstem. Based on our simulations we suggest that the closed-loop respiratory control system switches to active or forced expiration when a further increase of ventilation by a simple increase in the rate of breathing and phrenic amplitude (defining the maximal lung inflation) becomes insufficient or not effective enough to support gas homeostasis. In such cases, the control system activates the additional abdominal pump controlled by the late-E activity emerging in the RTN/pFRG that provides additional expulsion of the air from lungs by reduction of the minimal lung volume.

The major limitation of the present model results from a lack of peripheral chemoreceptor pathways, which do not allow us to explicitly consider their role in control of breathing. The model will be further extended by incorporating peripheral chemoreceptors and their pathways to study their role in closed-loop control of respiration under different conditions, including various respiratory disorders.

## Methods

### Parameters

All parameters in the model were based on studies on rats or tuned to match known observations from rats.

### Modeling the Pontine-Medullary Respiratory Network

The neuronal part of the model is an extended version of the model described by Rubin et al. [37]. The six neurons indicated in Figure 1 are modeled using a conductance-based description with non-spiking output. In this framework, the membrane potential 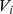 of neuron 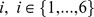, is governed by the equation
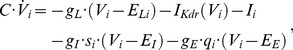
(1)where 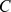 is a membrane capacitance, 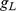 and 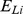 are the conductance and the reversal potential of the leak current, 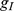,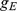 and 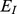,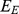 are the maximal conductances and reversal potentials of inhibitory and excitatory synaptic inputs, and 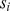,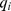 are the gating variables of inhibitory and excitatory synaptic channels, respectively.

The potassium rectifier current is described as
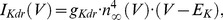
(2)where 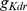 is its maximal conductance, 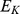 is the potassium reversal potential and
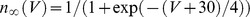
specifies the voltage dependence of the potassium rectifier instantaneous activation. 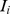 stands for a neuron-specific set of other ionic currents. For Pre-I/I and Late-E neurons (*i* = 1,5), 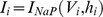, which is the persistent sodium current described as
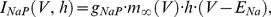
(3)where 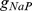 is its maximal conductance, 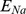 is the sodium reversal potential, 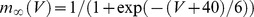 is the instantaneous activation, and 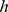 is the gating variable for inactivation. The dynamics of the inactivation variable 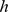 is governed by
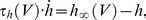
(4)where 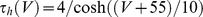 ms and 
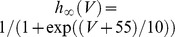
 are the voltage dependent time constant and steady state activation, respectively.

For Early-I, Aug-E and Post-I neurons (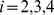), 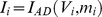, which is the potassium adaptive current given by
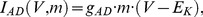
(5)where 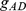 is its maximal conductance, 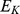 is the reversal potential for potassium, and 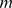 is the inactivation gating variable governed by
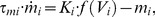
(6)where 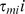 is a time constant, 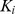 is a scaling parameter, and



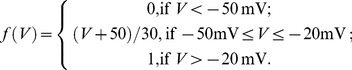
(7)is a function that represents the neuron’s activity level. For Ramp-I (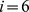), 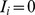.

The gating variables for synaptic conductances are derived from the activity of presynaptic neurons and other input sources using the equations
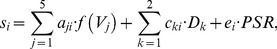
(8)

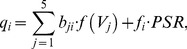
(9)where the function *f(V)* is defined in (7), the constant drive 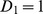 comes from the pons, the drive *D_2_* is due to the RTN as defined in (27) below, and the non-zero synaptic weights (*a, b, c, e, f*) are provided in Table 1.

**Table 1:** Parameters of neural network.

	Pre-I/I	Early-I	Aug-E	Post-I	Late-E	Pons	RTN	*PSR*
*i*	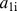	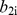	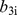	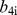	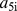	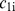	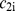	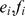
1: Pre-I/I			0.15	1.0	0.5	0.6	0.2	
2: Early-I	0.35		0.15	0.42		0.4	0.5	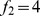
3: Aug-E		0.42		0.2	0.25		1.0	
4: Post-I		0.22				0.7		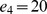
5: Late-E		0.075		0.12			0.15	
6: Ramp-I	0.35	0.3	0.7	0.7		0.25	1.1	

Note that *PSR* is the peripheral feedback provided by the lung stretch receptors. In our model, we use 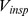, the inspired lung volume (excess of the lung volume above the basal volume, see (25)), as the *PSR* signal that is multiplied by the *e_i_, f_i_* values indicated in the table.

### Modeling the Lungs and Gas Transport

The lung and gas transport model represents an extended version of the model described by Ben-Tal and Smith [16] with certain parameters rescaled to represent the adult rat system. Diaphragm and abdominal effective displacements, 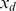 and 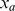, are described by
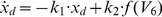
(10)

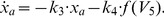
(11)These are dynamical variables that follow the activity of the ramp-I neuron, 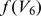, and the late-E neuron, 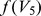, with the parameters 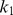 and 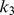
representing muscle recoil while 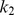 and 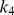 represent a conversion from neural activity to velocity.

The mechanics and gas exchange in the lungs are taken from [15], [39]. Specifically, diaphragm and abdominal muscles affect the pleural pressure 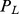 through the relationship
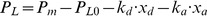
(12)where 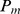 is the total pressure in the mouth, 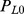 is the residual pressure, and 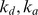 are the conversion coefficients. Note that 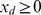 while 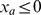. The alveolar pressure 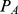 is a dynamical variable described by the differential equation
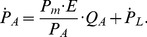
(13)Here 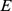 is the lung elastance and 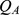 is the net flux of gas into the alveoli. This flux consists of the flux from (to) the mouth and diffusion to (from) the blood:
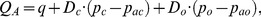
(14)where 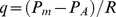 is the air flow from (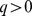, inspiration) or to (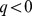, expiration) the mouth, with airway resistance 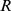. The terms concerned with diffusion in (14) include the diffusion capacities 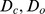, and differences of partial pressures in the blood 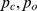 and alveoli 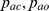 of carbon dioxide and oxygen, respectively. Alveolar partial pressures are expressed in terms of the relative content 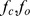 of carbon dioxide and oxygen in the alveoli:
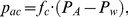
(15)

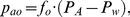
(16)where 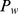 is the water vapor pressure at 38.5°C. Partial pressures of CO_2_ and O_2_ in the blood, 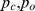, are dynamical variables that are reinitialized every heart beat (i.e. every 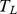 seconds, see parameter values in Table 2 below) to the predefined values 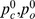; between heart beats, they obey the following equations:
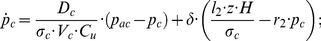
(17)

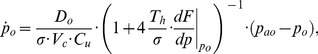
(18)where 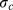 and 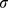 represent solubility of CO_2_ and O_2_ in blood plasma, respectively, 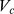 is the volume of the lung capillaries (assumed to be the same as the heart stroke volume), 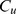 is the molar gas volume at 38°C and 760 mmHg, and 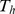 is the concentration of hemoglobin. In (17), *z* represents the concentration of bicarbonate in the blood; it is a dynamical variable reinitialized after each heart beat to 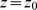 and between heart beats it is governed by the differential equation
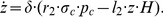
(19)where 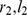 are the dehydration and hydration reaction rates, 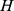 is the concentration of H^+^, and 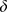 is the acceleration rate of the chemical reaction in which bicarbonate (HCO3-) binds with H+ and H2O and CO2 are released inside of a red blood cell. In (18), 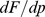 is the derivative of the hemoglobin saturation function
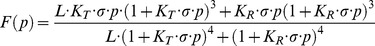
(20)with parameters 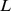, 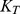, and 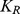 provided in Table 2 below.

**Table 2:** Parameters of the model.

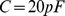	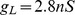	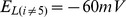	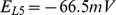
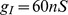	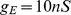	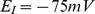	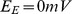
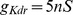	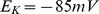	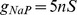	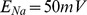
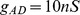	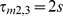	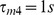	
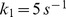	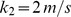	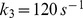	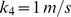
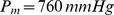	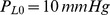	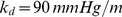	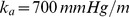
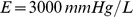	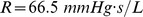	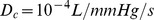	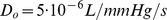
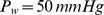	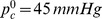	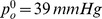	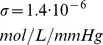
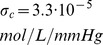	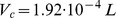	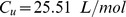	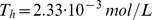
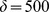	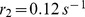	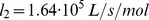	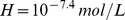
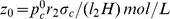	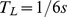	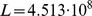	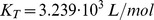
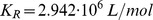	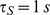	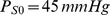	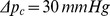

The dynamics of the relative content of gases in the alveoli is described as follows:
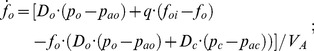
(21)

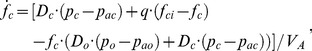
(22)where 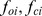 are inspired concentrations of oxygen and carbon dioxide, respectively, defined by
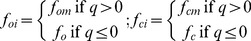
(23)

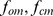
 are the relative concentrations of O_2_ and CO_2_ in the mouth, and 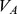 is the lung volume given by
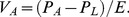
(24)


### Mechanoreceptor and Central Chemoreceptor Feedback

The activity of the pulmonary stretch receptors *PSR* was taken to be equal to the inspired lung volume:
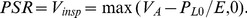
(25)Chemoreceptor activity (RTN drive) was modeled as a saturating function of the smoothed partial pressure 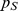 of carbon dioxide in the blood. To this end, we used the equation
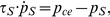
(26)where 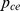 is the value of the CO_2_ partial pressure right before the most recent heart beat. The RTN drive was calculated using a simplified version of the expression suggested in [32],
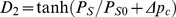
(27)where 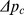 is used to simulate RTN stimulation where applicable (see text) and is otherwise set to 0.

All model parameters for the lungs and gas transport are shown in Table 2.

### Simulations

All simulations were performed using a custom simulation program written in C++. Differential equations were solved using the exponential Euler integration method with a time step of 0.1 ms.
